# Process Advantages of Direct CO_2_ to Methanol Synthesis

**DOI:** 10.3389/fchem.2018.00446

**Published:** 2018-09-27

**Authors:** Dana S. Marlin, Emeric Sarron, Ómar Sigurbjörnsson

**Affiliations:** Carbon Recycling International, Kópavogur, Iceland

**Keywords:** carbon dioxide utilization, emissions to liquids, green methanol, CO_2_ to methanol, industrial processes

## Abstract

Developing a laboratory scale or pilot scale chemical process into industrial scale is not trivial. The direct conversion of CO_2_ to methanol, and concomitant production of hydrogen from water electrolysis on large scale, are no exception. However, when successful, there are certain benefits to this process over the conventional process for producing methanol, both economic and environmental. In this article, we highlight some aspects that are unique to the process of converting pure CO_2_ to methanol. Starting from pure CO_2_ and a separate pure source of H_2_, rather than a mixture of CO, CO_2_, and H_2_ as is the case with syngas, simplifies the chemistry, and therefore also changes the reaction and purification processes from conventional methanol producing industrial plants. At the core of the advantages is that the reaction impurities are essentially limited to only water and dissolved CO_2_ in the crude methanol. In this paper we focus on several aspects of the process that direct conversion of CO_2_ to methanol enjoys over existing methods from conventional syngas. In particular, we discuss processes for removing CO_2_ from a methanol synthesis intermediate product stream by way of a stripper unit in an overhead stream of a distillation column, as well as aspects of a split tower design for the distillation column with an integrated vapo-condenser and optionally also featuring mechanical vapor re-compression. Lastly, we highlight some differences in reactor design for the present system over those used in conventional plants.

## Introduction

The carbon captured in the deposits of fossilized vegetation, along with ancient marine life, degraded and stored in the earth's crust, have been pivotal to human population growth and development, and indeed, have become essential to our existence (Richardson et al., 2011). Not only are these deposits finite, but their depletion represents a reversal of atmospheric composition to a time when atmospheric CO_2_ concentrations were much higher, and a regeneration of a climate that much of the present-day biosphere is not accustomed to (Letcher, 2016; Kondratyev and Cracknell, 1998; Akhtar and Palagiano, 2018). Global climate change is perhaps the most pressing environmental issue of our time (Princiotta, 2011; Letcher, 2016). The Intergovernmental Panel on Climate Change (IPCC) cites unequivocal scientific evidence for warming of the climate system (IPCC, 2014). A pivotal point in human history is upon us where we can choose to change the course we are on and embark on a more sustainable one. One approach to a more sustainable future is to use CO_2_ as the carbon source for fuels and carbon-based materials that are currently only derived from coal, oil and natural gas (Peters et al., 2011; Aresta et al., 2016; Landälv, 2017; Artz et al., 2018). Such an approach may have the dual effect of both removing CO_2_ already in the atmosphere and recycling and reusing what is emitted during combustion, thereby forming a static CO_2_ loop (Rahman et al., 2017).

Mitigating climate change is multifaceted and there are several viable options available for cleaner energy (Inui et al., 1998; Shindell et al., 2017). Solar and wind energy production, for example, has increased significantly in recent years, but still suffers from fundamental engineering limitations; the two main ones being storage and transport. One obvious way of storing and transporting electricity is by converting it into chemical energy (Peters et al., 2011; Landälv, 2017; Schemme et al., 2017).

Converting CO_2_ into methanol, which may be used as a fuel or fuel additive or precursor for more complex transportation fuels, or even as an intermediate for a diverse array of industrial chemicals including plastics, paints, textiles, and other uses, is an effective alternative disposition for CO_2_ (Olah et al., 2006; Gnanamani et al., 2014). By virtue of its physical properties, hydrogen bonding in particular, methanol is a liquid at room temperature and has much easier storage and transport capabilities than alternatives such as methane or hydrogen. Renewable methanol, wherein renewable electrical energy is converted to chemical energy and stored in the chemical bonds of methanol, provides a means of exporting energy from isolated places like Iceland with abundant renewable electricity, but having no electrical connection to neighboring countries. In addition, given the massive global methanol demand–200,000 tons per day and growing—diverting CO_2_ from the atmosphere and into methanol has not only significant potential for growth as a commodity chemical or fuel, but also the potential to recycle a large quantity of atmospheric CO_2_ (Alvarado, 2016).

Conventionally, methanol is produced on industrial scale from synthesis gas “syngas,” which is a combination of varying amounts of H_2_, CO, and CO_2_ frequently derived from gasified coal or natural gas (Ott et al., 2012; Sheldon, 2017). Processes for synthesizing methanol from syngas typically entrain CO and CO_2_, as well as, many other light and heavy weight coproducts along with the methanol product. The coproducts are a result of the more complex series of reactions that take place when the three reactant gasses interact with each other and the catalytic surface. (Olah et al., 2006; Hansen and Højlund Nielsen, 2008) Much of the subsequent energy and cost in conventional methanol plants is directed to these coproducts since they must be separated from the methanol product prior to the final disposition of the product.

**Figure 1:**
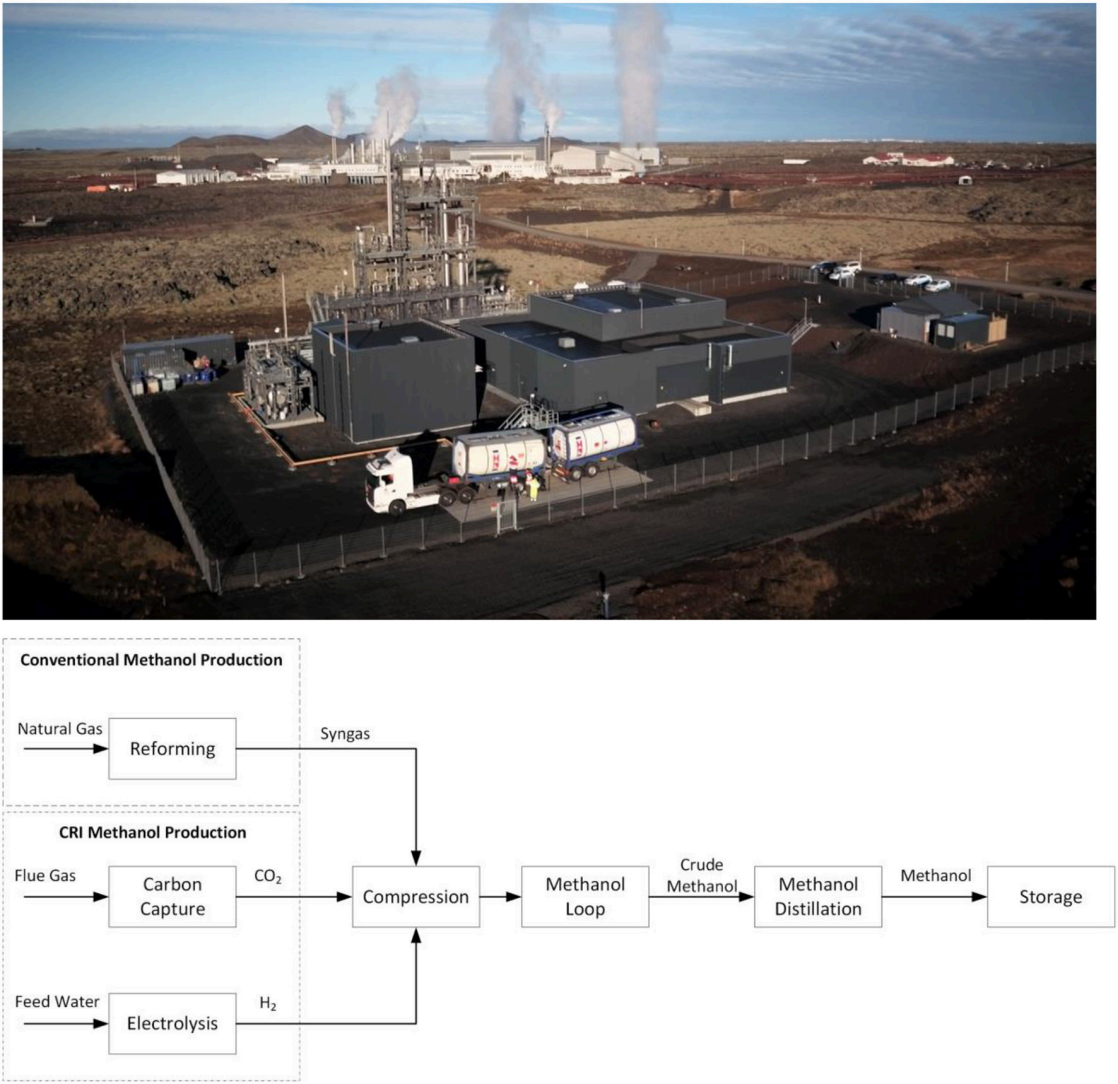
**(Top)** CRI's George Olah Renewable Methanol plant in Svartsengi, Iceland. **(Bottom)** Block flow diagram showing the different origins of syngas for the conventional process compared to the CRI process starting from CO_2_ pointing out the energy intensive reforming process in the former.

In this paper we offer an analysis from an industrial point of view on the main differences and advantages in the reaction and purification sections of a process for direct CO_2_ conversion to CH_3_OH, by comparison with the analogous process starting from syngas. Our remarks are based on our experience in the design and operation of the industrial scale methanol plant, Carbon Recycling International (CRI). CRI, located in Iceland, has operated the industrial scale direct conversion of CO_2_ to renewable methanol since 2012 (Figure 1). The plant is named in honor of the Nobel Prize laureate George Olah and has a capacity of 4,000 tons per annum of methanol. The CO_2_ is extracted and purified from the flue gases of the nearby geothermal power plant, while the hydrogen required for the production is generated by alkaline water electrolysis using Iceland's entirely renewable grid electricity.

## Discussion

Methanol synthesis starting from pure sources and controlled concentrations of CO_2_ and H_2_, greatly simplifies the chemistry and reaction products. In essence, the chemistry is reduced to only the following two reactions:

$$\begin{array}{l} {\text{CO}}_{2}+\text{​}3{\text{H}}_{2}\;⇌{\text{CH}}_{3}\text{OH}+\text{​}{\text{H}}_{2}\text{O }\Delta \text{H​}=\text{ ​}-49.16{\text{kJmol}}^{-1}(\text{Reaction 1})\\ \;{\text{CO}}_{2}+\text{​}{\text{H}}_{2}\;⇌\text{ CO}+\text{​}{\text{H}}_{2}\text{O }\Delta \text{H​}=\text{ ​}41.22{\text{kJmol}}^{-1}(\text{Reaction 2}) \end{array}$$

Several aspects of the process for producing methanol from syngas are different from those needed when using a pure source of CO_2_ and H_2_. For example, the formation of methanol from CO is based on CO reacting directly with H_2_ to produce CH_3_OH; the conversion is significantly more exothermic at−90.77 kJ/mol relative to Reaction 2 above (Ott et al., 2012). The main concern in process and reactor design is the removal of the heat of reaction. As a result, traditional methanol plant main reactors necessarily include an external coolant system, or stage conversion systems with intermediate cooling in the form of quenching or cold-shot gas injection (Hansen and Højlund Nielsen, 2008). The reactors used in methanol synthesis from syngas are typically limited to boiling water reactors (BWRs) due to the high heat profile of typical reaction suites as mentioned above. BWRs are complex and expensive equipment, but are typically necessary to mitigate the heat generated from the exothermic production of methanol from syngas to protect the reaction product, the reactor, and the catalyst. By contrast, methanol synthesis starting from pure CO_2_ by way of Reaction 1 may take place in a modified reactor due to the less intense exotherm compared to syngas reaction suites, enabling the use of tube-cooled reactors as the primary reactor. The use of a tube-cooled reactor is also advantageous over existing reactor configurations in terms of the lower cost, higher efficiency, and relative simplicity of operation. Additionally, tube-cooled reactors are preferred as being more efficient than adiabatic or cold-shot reactors which may require multiple reactors in series to achieve desired conversion rates. Further, improving the heat distribution with the reactor helps to prevent catalyst sintering, thereby extending the life of the catalyst and minimize process interruptions.

Regarding the purification of the crude methanol, having multiple gas impurities as well as liquid coproducts certainly complicates the purification steps needed. The condensate obtained from the loop is mainly a mixture of methanol and water known as crude methanol. The separation of water and methanol is relatively straight forward; however, the crude methanol produced in either traditional or CO_2_-based methanol loops also contains by-products and dissolved gases (especially CO_2_) which must be removed. Commonly occurring by-products include higher alcohols (mostly ethanol), esters, ethers (such as dimethyl ether), and ketones (such as acetone and methyl ethyl ketone). Ketones, in particular, pose an acute problem owing to their tendency to concentrate in the methanol product (Hansen and Højlund Nielsen, 2008). The concentration of these by-products has been shown to be highly dependent on CO concentration (Lorenz et al., 2014). Therefore, when production proceeds from CO_2_ rather than CO, a dedicated light-ends separation process can be designed that takes advantage of the significant reduction in byproducts.

A typical process in conventional methanol synthesis for removing gases such as CO, CO_2_ and high-boiling components such as ketones, usually entails separate and expensive fractionation column(s) and associated separations equipment and energy consumption in addition to the primary distillation column. Energy is consumed in both a reboiler and a condenser for the fractionation operation. The light-ends fractionation process is necessary because of the high solubility of CO_2_ in methanol, which cannot therefore be adequately separated by a simple flash operation. The separation of CO, CO_2_ and high-boiling components such as acetone from the desired methanol product is, as a result, highly expensive and inefficient. Contaminants with similar or higher boiling points than methanol pose an addition set of complications that are addressed with a heavy ends distillation column system. Heavy contaminants are not produced to any significant amount when limited to only Reactions 1 and 2, simplifying the separation of bottoms to essentially H_2_O. In addition, the use of pure CO_2_ as a starting material provides an alternative disposition for CO_2_ emissions, and facilitates a simpler and more efficient separations process, as will be described herein. Some details on the reaction processes and purification are given in the sections below.

## Reactor design for direct CO_2_ hydrogenation

Reactor design in methanol synthesis and in any chemical process is important for preserving catalyst life, for achieving acceptable production rate and quality, and for controlling process conditions. Existing methanol synthesis facilities typically comprise BWRs which are expensive and complex but are required to handle large temperature peaks due to the exothermic nature of methanol synthesis. Alternative reactors in existing methanol synthesis facilities typically comprise adiabatic or cold-shot reactors, which are less expensive than boiling water reactors but are inefficient and require the use of multiple reactors to achieve acceptable conversion rates. Existing facilities typically require a plurality of reactors, whether they are boiling water reactors, or adiabatic reactors, or a combination of both reactors.

In addition, existing reactor designs, because of the high temperatures normally present in methanol synthesis and other exothermic processes, further experience problems with catalyst sintering, wherein the normally crystalline catalyst reverts to its agglomerate state due to the high heat. Sintering reduces the effective life of the catalyst, leading to increased costs as the facility and process must be interrupted to allow for the catalyst to be removed, regenerated or replaced. Alternatively redundant reactor systems must be installed to allow for catalyst swap out without shutting down the facility. In extreme cases if reaction heat is not effectively removed, the resulting temperature increase can lead to reaction conditions where methanation reactions further increase the reactor temperature, which can lead to a loss of control of the process. Such runaway reactions are much less likely to occur with the direct CO_2_ hydrogenation reaction due to the lower reactivity and exotherm.

Also, existing reactor designs often comprise a hollow section in the center of the reactor which is used to structurally support the weight of the catalyst, e.g. by providing additional mechanical structures or supports. This unfortunately has the effect of unevenly distributing the catalyst, reactants, and cooling tubes (in tube cooled reactors).

As described above, the problems with existing reactors are largely mitigated when synthesizing methanol by only Reactions 1 and 2, which results in a lower heat profile than existing methanol synthesis reaction suites. Because of the lower heat profile, a boiling water reactor is not required in order to control the temperature of the reactor. A tube-cooled reactor alone is generally sufficient to control the temperature resulting from Reactions 1 and 2. Moreover, a single tube-cooled reactor is sufficient to produce the desired methanol product as it is more efficient than adiabatic reactors due to its lower operating temperature, and multiple reactors in series are not required for conversion. Thus, by limiting only to Reactions 1 and 2, a single tube-cooled reactor may advantageously be used to achieve desired methanol production. In a tube-cooled reactor, multiple tubes are added to the catalyst support plate. The addition of tubes helps to distribute heat more effectively and consistently in the reactor. The more even heat distribution prevents hot spots and thus minimizes catalyst sintering, thereby extending the useful life of the catalyst between regenerations.

## Gas stripper for removal of light ends

Because all distillation processes require energy input through the reboiler and condenser to achieve separation of the components, and therefore produce emissions, it is highly beneficial to have a separations section that minimizes the number of distillation operations required to achieve desired separation of the components, and consequently minimizes equipment and operating costs, as well as emissions. Column distillation accounts for 40–60% of the energy used by the chemical process industry, equivalent to at least 1.2 million barrels of crude oil per day (Kiss et al., 2012). In general terms, distillation alone accounts for 6% of total U.S. energy use. The massive energy consumption of distillation operations is at least partly due to the inefficiency of existing distillation processes which operate with approximately 5–10 % efficiency (Kiss et al., 2012).

Existing methanol synthesis operations typically comprise a column that is dedicated to separating light components (meaning components having a lower boiling point than the methanol product), and consequently generates emissions. Additional complications arise when light end impurities form azeotropic mixtures with methanol, such as acetone, ethyl formate, methyl acetate, and methyl propionate, with the primarily observed azeotropic impurity typically being acetone. Azeotropic mixtures are extremely difficult to separate, and essentially affect product recovery since azeotropic mixtures in a light ends column increases the concentration of methanol in the vent. Consequently, a high concentration of light impurities in the crude leads to unavoidable losses of product methanol, and conversely, lower impurities results in higher product recovery. In some cases, azeotropic mixtures may also require special separations techniques and equipment such as pressure swing distillation or addition of another chemical species.

**Figure 2:**
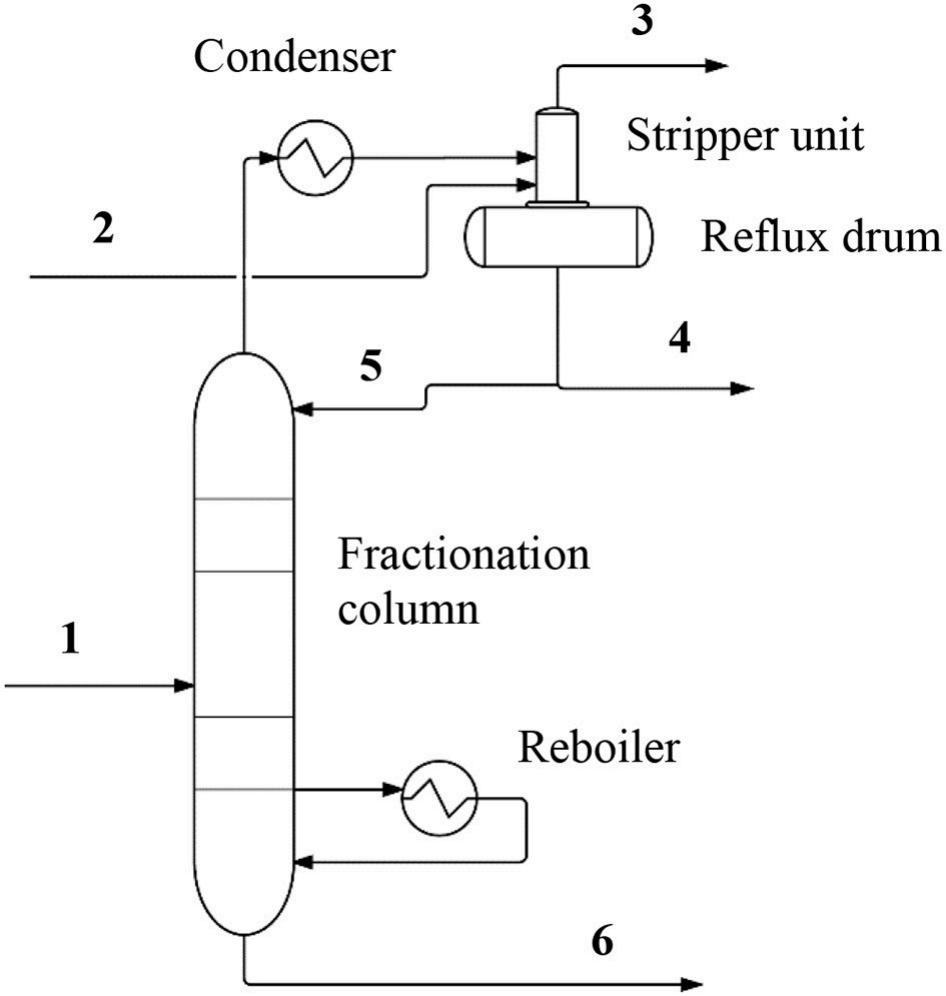
A simplified diagram of a separations system showing the reactor and the gas sparger unit. Process and component labels are included, and the streams are identified by numbering as follows (select conditions are provided where appropriate): (1) Feed approximately 50% methanol (molar); (2) Carrier gas (composition described in text); (3) Waste gas (mainly CO_2_ and carrier gas); (4) Methanol product; (5) Reflux stream; (6) Lean methanol (composition).

In contrast, for Reactions 1 and 2 effective purification of the methanol product may be achieved by a single column-separation followed by a stripping operation, without the need for a dedicated light ends column to further separate the desired product from impurities in the condensed overhead stream, or a heavies column to remove heavy oil impurities, or other special separations techniques. The system for conversion and separation of gases includes a reactor producing a crude product stream and a fractionation column connected at an overhead section to a heat exchanger and a stripper unit (Figure 2). A carrier gas stream (2) is bubbled through the stripper unit to selectively remove impurities such as CO_2_ from the overhead stream of the fractionation column after the stream has been condensed in the heat exchanger. Variations of the stripper unit may include a gas sparger unit integrated with a reflux drum, the reflux drum being configured to receive the condensed overhead stream from the heat exchanger. The gas sparger unit flows a stripping fluid, such as N_2_ or H_2_ gas, through the condensed overhead stream in the drum to selectively remove CO_2_ from the methanol product without the use of a dedicated separation column. A variety of stripping fluids may be used. The system may thus be adjusted to the specific configuration of products and reactants needed and available.

In a typical system, reactor effluent contained in the crude methanol stream is fed to separations section which comprises fractionation column and accompanying equipment. Owing to the composition of crude methanol, the stream contains virtually no CO or other light contaminants besides water and CO_2_, and therefore, only a single fractionation column is required for purifying the desired methanol product. This is a singular advantage over most methanol purification processes which comprise dedicated light ends columns and associated equipment such as reboilers, condensers and drums in order to achieve adequate purification of the product. The condenser and reboiler provide the required duty for the fractionation column to separate the crude methanol stream into on-spec products.

Furthermore, in the case where the reactor primarily consumes CO_2_ as a feedstock for methanol synthesis, a viable alternative disposition for CO_2_ emissions is available and recycling of the removed CO_2_ to the reactor generates a continuous loop that may ultimately convert all the CO_2_ into methanol. The integrated stripper/reflux drum drives the CO_2_ out of the condensed overhead stream by introduction of a suitable carrier gas. The gas sparger typically introduces the carrier gas by bubbling the carrier gas up through the collected liquid within the reflux drum portion of the integrated stripper/reflux drum. The combined carrier gas and CO_2_ are ejected from the integrated stripper/reflux drum in the form of a waste gas stream (3) that may be disposed of in various ways; for example, waste gas stream may be scrubbed of entrained methanol in a scrubber unit prior to being released to the atmosphere or, recycled/reprocessed as additional feedstock to the reactor, or disposed directly to battery limits or another process. The stripper unit may also be arranged either upstream or downstream of a reflux drum, as determined by the process requirements of a specific configuration or facility.

## Split tower arrangement

Distillation is a highly energy-intensive process because of the inherent inefficiency of the separation process, which requires large duties in both the reboiler and condenser, as well as significant reflux rates to achieve desired separation. In facilities or processes where substantial heat is left over from initial reaction units (due to reaction or thermal inefficiencies, or high reaction exotherms), or from associated processes at the same facility or site as the separation process, the leftover heat, often being in the form of generated steam, may be used to provide duty to the reboiler of certain of the distillation operations. However, such an arrangement may be undesirable in the first instance, as the generation of substantial waste heat in the reaction phase or in associated processes represents a thermodynamic inefficiency and consequently a negative effect on emissions from the facility. It is therefore desirable to minimize the generation of waste heat as much as possible. It is also desirable to minimize heat requirements of processes located downstream of the reaction process which may otherwise utilize waste heat, such as in reboilers. By so doing, requirements for added heat may be reduced.

One approach to improving heat utilization efficiency is with a split column system wherein the bottom section of the column may operate at a higher pressure than the top section of the column. The reflux of the bottom section of the column may be integrated with the reboiler of the top section of the column, utilizing a single heat exchange device to reduce the total duties for the fractionation operation. The energy savings realized through this arrangement further contributes to emissions reductions. Additionally, the heat exchange device may optimize temperature approach (and as a result enhance thermodynamic efficiency) between the integrated reboiler and condenser streams and thus between the two columns by utilizing a falling-film evaporator or thermosiphon design. The improved (lower) temperature approach of falling-film evaporator- and thermosiphon-type heat exchangers compared to, for example, the temperature approach of kettle reboilers, may enable a lower operating pressure in the higher-pressure column, as the pressure required for the overhead stream to provide sufficient reboiler duty for the lower pressure column is reduced. This also minimizes capital and operating costs (because the required reboiler duty is reduced and the column itself may be reduced in size), which puts further downward pressure on emissions.

For the synthesis of methanol from CO_2_ in geothermal steam, and utilizing the heat derived from the process, as is the case for Carbon Recycling International in Iceland, the efficiency of heat use and reuse becomes a crucial factor in the overall efficiency of the process. Although a split tower arrangement is not unique to this process, there is certainly an economic advantage when used at this scale. For instance, a typical reformer-based methanol plant produces excess steam and there is little incentive to optimize heat reuse. A CO_2_ based process using renewable or geothermal energy, where heat generation is at a premium, must have tighter energy use requirements, and a split tower arrangement becomes essential, both because it lowers energy use and because it lowers the CO_2_ footprint.

**Figure 3:**
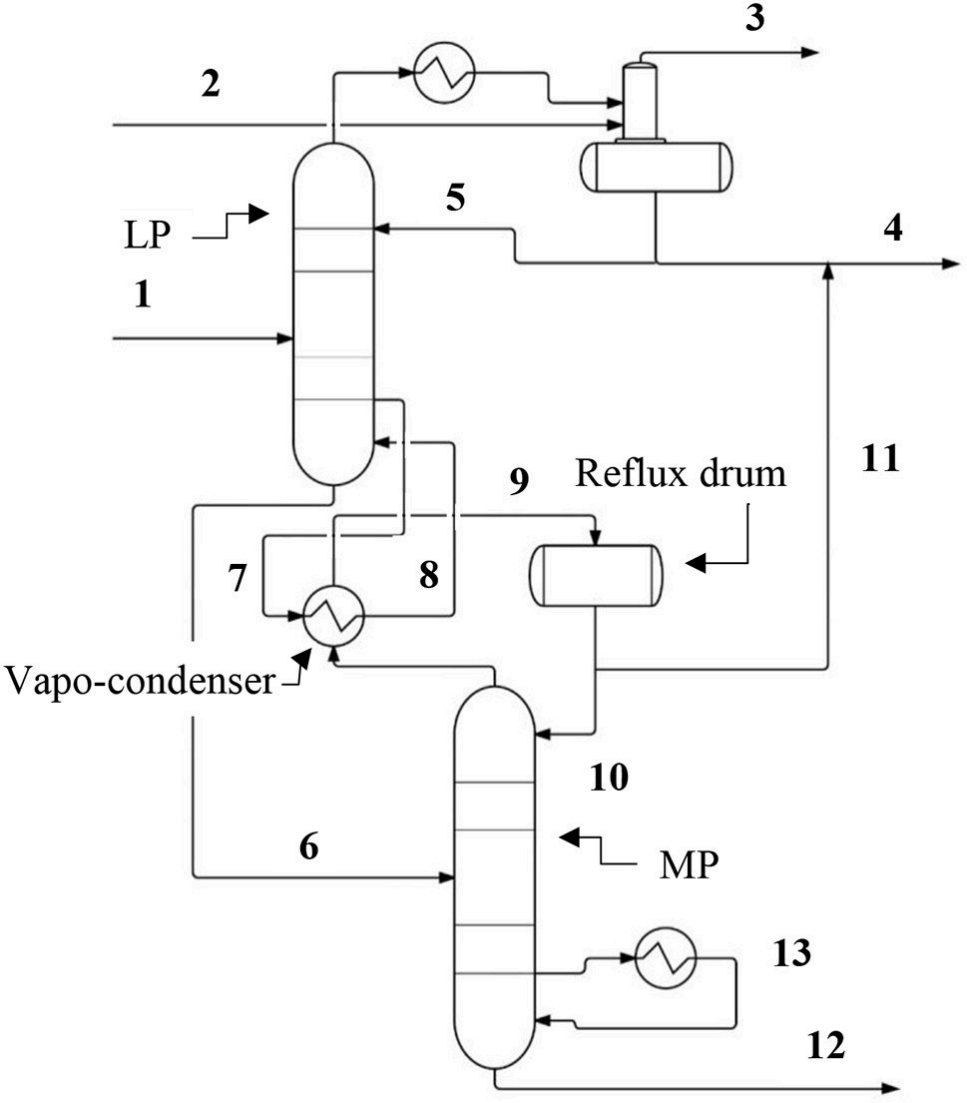
A simplified diagram of the split column separations systems. Process and component labels are shown where they have not already been identified in Figure 2. The streams are identified by numbering as follows: (1) Feed comprising approximately 50% methanol (molar); (2) Carrier gas; (3) Waste gas (CO_2_ and carrier gas); (4) Methanol product; (5) Reflux stream; (6) MP stream enriched in water; (7 and 8) LP column reboiler stream; (9 and 10) MP column reflux stream; (11) Methanol product; (12) Bottoms (waste water); (13) MP column reboiler stream.

In a simplified system as shown in Figure 3, the methanol stream is fed to separations section, which comprises a fractionation column. The fractionation column is arranged in a split tower arrangement comprising a top or low pressure (LP) section and a bottom or medium pressure (MP) section. The LP section and MP section are connected by the stream which comprises primarily methanol and water, as well as by heat integration between the condenser of MP section and the reboiler of LP section. A Vapo-condenser unit integrates the functions of both condensing an overhead stream of the MP section and re-boiling a bottom stream of the LP section. The operating pressure of the MP section is calibrated to be sufficiently high such that the condensation of the stream provides the required duty to re-boil the stream derived from the LP section. The use of the split tower arrangement enhances thermodynamic efficiency by reducing total duty required in the fractionation column, as the duties that are integrated in the vapo-condenser between the two sections would otherwise be provided in a condenser at the overhead of MP section and a reboiler at the bottom of LP section, or in increased duties to the LP section condenser and the MP section reboiler.

As with the case above in Figure 2, an overhead stream made up of CO_2_ and methanol from the LP section is fed to a condenser and then to an integrated stripper/reflux drum, while the MP section separates the contents of the lower stream into product methanol and waste water. Substantially no CO_2_ is entrained in the stream entering the MP section because the CO_2_ contained in the crude methanol stream entering the LP section is isolated in the overhead stream of the LP section. A reboiler provides additional duty at a bottom portion of the MP section. Waste water product is obtained at the bottom of the MP section and disposed of. A reflux drum receives the condensed overhead stream of MP section, and splits the condensed overhead stream into a reflux return stream which is returned to the MP section, and a MP section methanol product stream which is combined with the LP section methanol product stream. The combined methanol product stream can be sent to battery limits, storage, or to another disposition.

## Mechanical vapor recompression

In certain applications, available heat and/or steam (which is normally used to provide reboiler duty) is limited; for example, certain processes, such as in geothermal processes, may not have a high exotherm and thus do not produce significant amounts of waste heat for steam generation which can be used to provide heat to other processes. In such applications, and in distillation generally, a separations section which minimizes heat requirements, such as in reboilers, is desirable to avoid the costs and emissions associated with steam generation to make up for heat recovered from hot sections of the process. Additionally, it is desirable to minimize capital costs by reducing the size and number of units required to carry out a separation operation.

In variations of the split column process, the split column may utilize mechanical vapor recompression (MVR) to further improve thermodynamic efficiency by taking a side cut from the top section of the column, recompressing the side cut, and then feeding the compressed side cut to the bottom section of the column. This advantageously improves the efficiency of the separation by substituting the increased temperature and pressure resulting from compressing the side cut for the duty that would otherwise be required of a dedicated reboiler for the bottom section of the column. This arrangement thereby also reduces capital costs and operating costs.

**Figure 4:**
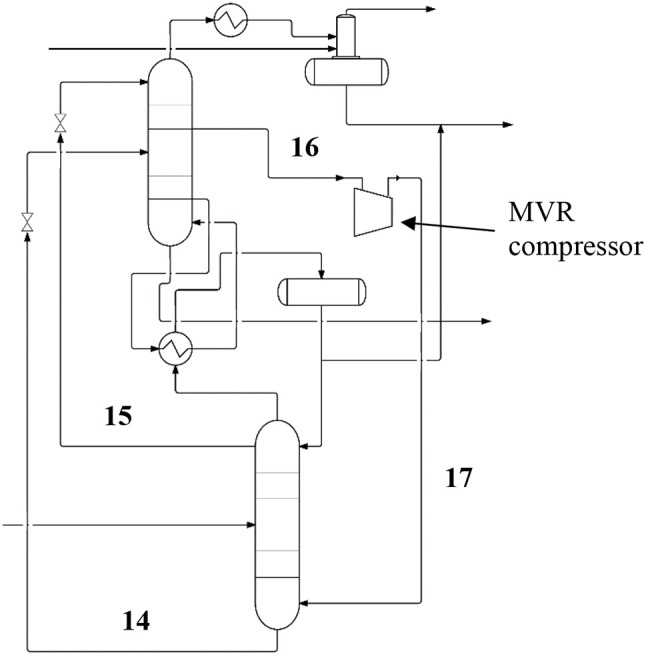
A simplified diagram showing the complete system featuring the reactor and the gas sparger unit, split column arrangement, and the mechanical vapor recompression unit. The labels and stream flows identified in Figures 2, 3 apply to this diagram and are omitted for clarity. The additional process labels relating to the mechanical vapor recompression unit are included here and the additional stream flow numbering labels are as follows: (14) Lean methanol; (15) Rich methanol reflux to LP; (16 and 17) Recycle stream.

To illustrate its operation, the MVR unit is integrated in the separations section as shown in Figure 4. The crude methanol stream containing methanol, water, and CO_2_ is fed to the fractionation column, which is arranged in a split tower arrangement similar to the embodiment of Figure 3. The top or LP section is heat integrated with the bottom or MP section at a vapo-condenser which condenses an overhead stream of the MP section and re-boils a bottom stream of the LP section. The crude methanol stream is received at an optimal location in the MP section. The LP section and MP section are further connected by streams originating from the lower and upper sections of the MP section, which function to provide material balance and reflux for the LP section if needed, thereby eliminating the need for a separate reflux stream from an overhead stream of LP section. Waste water product is removed from the bottom of LP section.

As with the previous two systems, a condenser condenses an overhead stream of the LP section and feeds the condensed overhead stream to an integrated stripper/reflux drum. The CO_2_ and carrier gas are scrubbed of entrained methanol prior to atmospheric release or recycled/reprocessed as additional feedstock for the reactor. Because the direct streams from the MP to the LP sections provide reflux from the MP section, no reflux stream needs to be returned to the LP section from the LP section overhead stream. As a result, the required flowrate of the LP section overhead stream is reduced and the required size of condenser at the top of the LP section, as well as duty removed therethrough via cooling water, is consequently greatly reduced. This further reduces capital and operating costs, as well as emissions.

A sidecut from the LP section is fed to a MVR compressor which compresses the sidecut to an operating pressure suitable for the MP section, thereby also raising the temperature of the sidecut. The recompressed sidecut is fed to the MP section, preferably at a location near the bottom of the MP section. The recompressed sidecut replaces the reboiler of the MP section, as the added enthalpy of the compressed sidecut serves to provide the necessary duty to reboil the MP section and consequently the LP section. The addition of this duty by the MVR compressor achieves enhanced thermodynamic efficiency and capital cost reductions compared to providing the duty through a reboiler unit.

Recompression of a stream may thus advantageously utilize compressor work to raise the pressure and consequently temperature of a portion of a process stream (such as a sidecut from the LP column section) for the purposes of providing reboiler duty more efficiently than adding heat to the process through a conventional reboiler, especially a reboiler utilizing steam as a heat source. Recompressing an existing vapor stream to a higher temperature and pressure using a compressor advantageously bypasses the phase change inefficiencies inherent in steam generation from boiler feed water due to the high enthalpy difference between boiler feed water and pressurized steam. Mechanical vapor recompression therefore attains the desired increase in temperature and pressure with a much lower input of energy than traditional reboilers.

The efficiency of the MVR is further enhanced by feeding the recompressed sidecut directly to a bottom portion of the MP section to replace the reboiler and the heat exchange inefficiencies associated therewith. The recompressed sidecut can more efficiently transfer heat to the MP section by interacting directly with the contents of the column. The use of mechanical vapor recompression thus solves the problem of distillation and other process operations requiring added heat, and leads to lower emissions and decrease in capital and operating costs.

## Summary

In this paper we put forward that there are both inherent advantages and disadvantages to producing methanol directly from separate sources of CO_2_ and H_2_, and that overall, it is an advantageous, cleaner, less energy intensive and more environmentally friendly process than conventional processes using a fossil fuel based syngas. We highlight several process advantages over the conventional methods, and offer ways to enhance the efficiency of industrial conversion of CO_2_ and H_2_ to methanol.

Typical disadvantages associated with CO_2_-to-methanol may include that CO_2_-syngas is less reactive than CO-syngas, which may then lead to larger reactors being needed. In addition, more water is typically produced in this reaction due to the reverse water gas shift as a consequence of the higher CO_2_ partial pressure. Lastly, largely for the same reason, there is typically more CO_2_ in the crude methanol.

Among the advantages, however, are that the CO_2_-to-methanol reaction is inherently more selective and results in fewer byproducts, and the reaction conditions are milder due to less exothermic reaction. Furthermore, there is an inherently better carbon utilization compared with conventional syngas. In a process where CO_2_ and H_2_ are added in separate pure streams, and independent of each other, the ideal stoichiometric ratio, depending on the catalyst requirements, may be adjusted precisely without the need for expensive reforming equipment. This is not the case when the syngas is generated as a mixture of gasses as in the conventional processes. Lastly, CO_2_ recycling will ultimately be beneficial for mitigation of global CO_2_ emissions and consequently, global warming.

Finally, some particular advantages of the CRI methanol plant include: (i) a reactor that makes uses of milder reaction conditions to function with a low-cost vessel design which, in turn, offsets the above-mentioned deficiency that the CO_2_-to-methanol pathway is less reactive, and (ii), catalyst selection that has been optimized for CO_2_ syngas conversion. Additionally, the efficient distillation design discussed herein allows that processes like CRI's methanol plant consumes less energy than conventional processes, and produces pure methanol at comparable cost even though the water content is higher.

## Author contributions

DM drafted the main body of the article. ES contributed to the technical aspects of the article. ÓS contributed to the overall structure and background of the article.

### Conflict of interest statement

All authors are employed by Carbon Recycling International.
